# Nutritional Interventions as Beneficial Strategies to Delay Cognitive Decline in Healthy Older Individuals

**DOI:** 10.3390/nu10070905

**Published:** 2018-07-15

**Authors:** Blanka Klímová, Martin Vališ

**Affiliations:** 1 Department of Applied Linguistics, University of Hradec Kralove, Rokitanskeho 62, Hradec Kralove 500 03, Czech Republic; 2 Department of Neurology, University Hospital Hradec Kralove, Sokolska 581, Hradec Kralove 500 05, Czech Republic; martin.valis@fnhk.cz

**Keywords:** nutrition, healthy older individuals, cognitive decline, intervention, prevention, randomized clinical trials

## Abstract

Current demographic trends indicate that the population is aging. The aging process is inevitably connected with cognitive decline, which manifests itself in worsening working memory, processing speed, and attention. Therefore, apart from pharmacological therapies, non-pharmacological approaches which can influence cognitive performance (such as physical activities or healthy diet), are being investigated. The purpose of this study is to explore the types of nutritional interventions and their benefits in the prevention and delay of cognitive delay in healthy older individuals. The methods used in this study include a literature review of the available studies on the research topic found in Web of Science, Scopus, and MEDLINE. The findings show that nutritional intervention has a positive impact on cognitive function in healthy older people. However, it seems that the interactions between more than one nutrient are most effective. The results reveal that specifically the Mediterranean diet appears to be effective in this respect. Moreover, the findings also indicate that multi-domain interventions including diet, exercise, cognitive training, and vascular risk monitoring have a far more significant effect on the enhancement of cognitive functions among healthy older individuals.

## 1. Introduction

Currently, the number of older people is rapidly increasing. For instance, in 2017 there were 962 million people aged 60+ years worldwide, with their number increasing by 3% every year. The largest proportion of the aging population can be found in Europe (25%). By 2050, all continents except Africa will have one quarter or more of their population aged over 60 years [1]. This demographic trend of aging population will cause significant social and economic problems [2]. Therefore, there are efforts to develop relevant strategies and guidelines both at national and international levels in order to delay this process of aging and prolong the active age of older individuals, as well as to maintain quality of life [3].

The aging process is inevitably connected with cognitive decline, which manifests itself in worsening working memory, processing speed, and attention [4], i.e., in so-called fluid intelligence, which deteriorates at different rate in each individual [5]. The rate and severity of cognitive decline varies from normal age-related change to chronic neurodegenerative diseases such as Alzheimer’s disease for which at present there is no cure [6]. Therefore, non-pharmacological approaches which can influence fluid intelligence, such as physical activities or healthy diet, are being investigated [6,7,8]. Evidence-based studies [9,10] indicate that specifically the Mediterranean diet (MedDiet) rich in olive oil and nuts, seems to contribute to the prevention of cognitive impairment among healthy older people. However, other food products, for example, avocados [11], or nutritional supplements such as consumption of flavanone-rich 100% orange juice [12] or omega-3 fatty acid [13] also play an important role in the prevention of cognitive decline in healthy older individuals. In addition, nutritional interventions in comparison with the pharmacological treatment, are usually well-tolerated, easy-to-implement, cost-effective, and safe for long-term use [13].

The purpose of this study is to explore the types of nutrition intervention and its benefits in the prevention and delay of cognitive delay in healthy older individuals.

## 2. Methods 

The authors conducted a literature review of the research studies on the basis of the key words in three acknowledged databases: Web of Science, Scopus, and MEDLINE. This review was performed over the period from 2014 to June 2018 since several review studies had been already written on this topic [14,15]. The key words were as follows: diet AND cognitive decline AND healthy older people, diet AND cognitive decline AND healthy elderly, nutrition intervention AND cognitive decline AND healthy older people, nutrition intervention AND cognitive decline AND healthy elderly, nutrition AND cognitive decline AND healthy older people, nutrition AND cognitive decline AND healthy elderly, diet AND dementia AND healthy older people, diet AND dementia AND healthy elderly, nutrition intervention AND dementia AND healthy older people, nutrition intervention AND dementia AND healthy elderly, nutrition AND dementia AND healthy older people, nutrition AND dementia AND healthy elderly.

**Figure 1:**
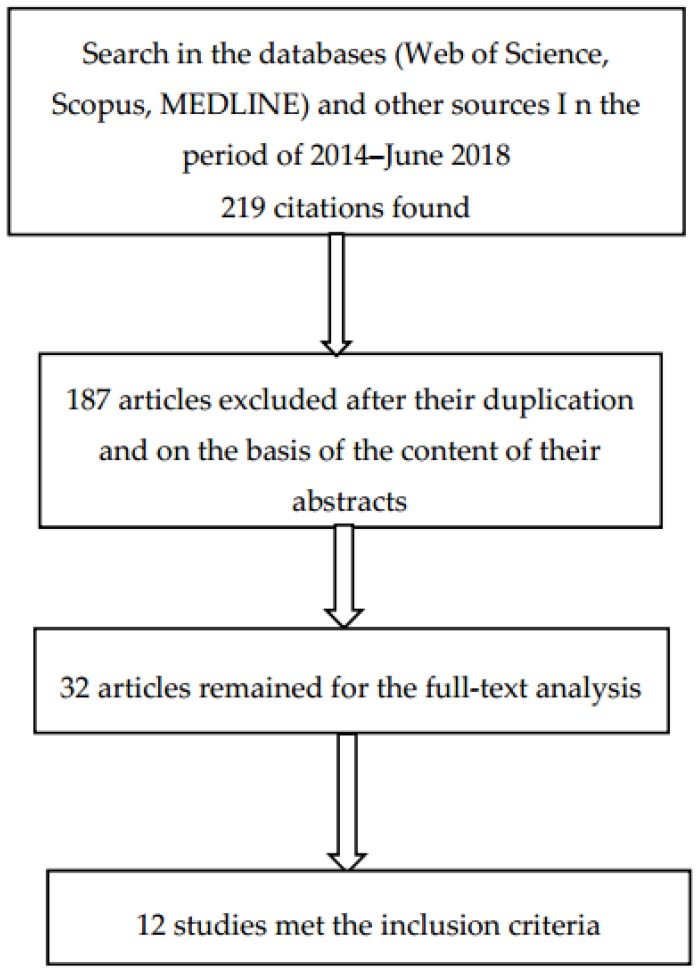
An overview of the selection procedure.

The majority of the studies were detected in the Web of Science database (97 studies), followed by Scopus (66 studies) and MEDLINE (45 studies). However, in MEDLINE it is possible to look only for clinical trials, and therefore the selection was easier. Altogether, 208 studies were found via the database search and 11 from other available sources (i.e., web pages, conference proceedings and books outside the scope of the databases described above). The titles of all studies were then checked in order to discover whether they focused on the research topic or not and irrelevant studies were excluded. In addition, the duplicate studies were also excluded. Afterwards, the authors checked the content of the abstracts whether the study examined the research topic. Thirty-two studies/articles were selected for the full-text analysis. Only 12 studies were then able to be used for detailed analysis of the research topic. The selection of these studies is described below (Figure 1).

The study was included if it was a randomized controlled trial, and matched the corresponding period, i.e., from 2014 up to June 2018. Furthermore, the study was included if it involved healthy older people, i.e., those without any cognitive impairment or dementia aged 50+ years, and focused on the research topic, i.e., nutritional intervention in the delay and prevention of cognitive decline in healthy older individuals. All studies had to be written in English. Thus, studies such as [5] were excluded, as well as cross-sectional descriptive and cohort studies, for example [16,17,18].

## 3. Results

Altogether 12 randomized controlled trials (RCT) were found. The majority of were from Europe (i.e., Finland, Germany, Italy, Spain, and the UK) [10,12,13,19,20,21,22,23,24] and the rest from the USA and Australia [11,25,26]. The nutrition intervention involved dietary supplements (i.e., cocoa flavanols, *Vitis vinifera*, docosahexaenoic acid (DHA)-rich fish oil, or omega-3 fatty acid), vegetable (e.g., avocado), berry and orange beverages, and the MedDiet. Two of the studies were multidomain lifestyle intervention studies including, apart from healthy diet, physical exercises, social activities, cognitive training, and vascular risk management [20,24]. The intervention period in the studies ranged from five weeks to five years. The subject samples also varied; the smallest sample of subjects consisted of 37 healthy older individuals and the largest included 775 healthy older people. The efficacy of the nutrition intervention focused on the prevention and delay of cognitive decline in the studies was measured with available validated cognitive assessment tools such as a battery of cognitive tests aimed at assessing working memory, attention, or processing speed. Table 1 below provides an overview of the main findings of these studies. They are summarized in alphabetical order of their first author.

Apart from one study [26], results of all RCTs indicate that the nutritional interventions had a positive impact on the cognitive performance of healthy older individuals, specifically on their working memory and attention [10,11,23]. Moreover, the findings of the multidomain lifestyle intervention studies reveal that such interventions exhibit significant impacts on cognitive functioning in later life [20,24]. The results also show that the change in the cognitive performance can be already detected after five weeks of intervention [23]. However, this is questionable because according to Kurz and van Baelen [27], the relevant period for medication assessment by regulatory authorities is 24 weeks at a minimum.

## 4. Discussion

The findings described in Table 1 show that nutrition intervention has a positive impact on cognitive functioning of healthy older people. The trials with the exception of four RCTs [10,19,23,26], concentrated only on one type of nutritional supplement (i.e., *Vitis vinifera*, docosahexaenoic acid (DHA)-rich fish oil, omega-3 fatty acid, avocado, and berry). Three RCTs [12,22,25] focused on high flavanol-rich drinks, which appeared to have quite a positive effect on cognitive performance among healthy older individuals. In fact, all nutrients described in Table 1 are part of the traditional multi-nutrient MedDiet pattern, and the research [28] indicates that the interaction of specific foods and nutrients, especially in the MedDiet, is more powerful on the aging brain than individual nutrients or a low-fat diet. In fact, the MedDiet seems to be a nutritional model for healthy dietary habits since it contains all the needed nutrients: monounsaturated fatty acids, polyunsaturated fatty acids, antioxidants (e.g., allium sulphur compounds, anthocyanins, beta-carotene-flavonoids, catechins, carotenoids, indoles, or lutein), vitamins (A, B1, 6, 9, 12, D, E), and minerals (magnesium, potassium, calcium, iodine, zinc, selenium) [28]. In addition, the combination of these nutrients positively affects pathological neurodegenerative processes such as oxidative stress, neuroinflammation, insulin resistance, and reduced cerebral blood flow [29]. Nevertheless, the MedDiet appears to improve cognitive performance in case there is a high adherence to this diet [30,31,32]. Furthermore, the MedDiet has a positive impact on neuropsychological [19] and physical state of older people [16] and on vascular diseases and diabetes [22,23,24,33]. Although the results mainly concerned western developed countries, similar research (however, non-RCT) was conducted among healthy older adults in Asian countries such as Taiwan [34] and Japan [35]. The findings of these studies also show a positive correlation between the healthy diet rich in vegetables, soy products, fruit, and fish and the prevention and delay of cognitive decline in older age. Wright et al. [36] in their study carried out among 2090 African Americans and whites emphasized that higher diet quality was associated with higher performance on tests of attention and cognitive flexibility, visuospatial ability, and perceptual speed. This was also evidenced by Smyth et al. [18] who in their cohort study claimed that higher diet quality is associated with a reduced risk of cognitive decline. Hosking et al. [37] report that adequate nutrition is essential for cognitive development in childhood and cognitive ability in childhood is strongly associated with cognitive performance across the lifetime.

Moreover, it seems that multi-nutrient dietary intervention should be implemented in the delay of cognitive decline among healthy older individuals. The multi-nutrient intervention is also one of the priorities discussed at the First WHO Ministerial Conference on Global Action against Dementia in March 2015 [38]. Furthermore, Shilsky et al. [39] suggest in their study that healthcare systems should play a more significant role in integrating nutrition care for healthy older individuals and that the nutrition assessment should be incorporated in the medical records. This was also confirmed by the discussed study [21], for which findings revealed that the dietary counselling had a positive impact on age-related diet quality and cognitive performance.

The findings of the reviewed RCTs [20,24] indicate that the multi-domain interventions including healthy diet, physical exercises, cognitive training, and vascular risk monitoring have a far more significant effect on the enhancement of cognitive functions among healthy older individuals [40]. In fact, these non-pharmacological strategies (healthy diet, physical exercises and cognitive training) have been confirmed by other research studies [7,8] and they should be all integrated at an optimal level into daily regime of healthy older individuals in order to prolong their active and quality life. This was also proposed at the International Conference on Nutrition and the Brain in Washington in 2013 [41].

On the contrary, research implies that dietary patterns rich in fat and sugar, with high intake of meat/poultry or eggs, have negative, harmful effects on cognitive functioning in older age [34,35,37].

The limitations of the reviewed studies consist in the small sample sizes, different types of nutrition interventions and outcome measures, and a lack of follow-up assessments, as well as the fact that not all studies were specifically designed to examine cognitive performance. All these insufficiencies might generate overestimated conclusions in this review study [42,43]. Therefore, more RCTs should be conducted to prove the efficacy of nutritional interventions on the prevention and delay of cognitive decline.

## Figures and Tables

**Table 1:** Overview of the twelve selected studies focused on cognitive decline and its prevention by nutrition intervention.

Author	Objective	Type of The Nutrition Intervention And Its Frequency	Intervention Period	Number of Subjects	Main Outcome Assessments	Main Findings
Brickman et al. [25] RCT (USA)	To investigate whether the enhancement of dentate gyrus (DG) function with dietary flavanols improves cognition in older adults.	Daily intake of 900 mg cocoa flavanols in the intervention vs. 10 mg cocoa flavanols in the control group.	Three months.	37 healthy older individuals, age: 50–69 years.	Functional magnetic resonance imaging (fMRI), a battery of cognitive tests, statistical analysis.	The results indicate that DG dysfunction is a driver of age-related cognitive decline and suggest non-pharmacological means for its amelioration such as the daily high flavanol intake.
Calapai et al. [19] RCT (Italy)	To investigate the potential beneficial effects of a *Vitis vinifera*-based dietary supplement on cognitive function and neuropsychological status in healthy older adults.	Cognigrape^®^ (250 mg/day) in the intervention group and placebo in the control group.	12 weeks.	57 subjects in the intervention group and 54 subjects in the control group; age: 55–75 years.	A battery of cognitive and neuropsychological tests, statistical analysis.	The findings reveal that 12 weeks of Cognigrape^®^ supplementation is safe, can improve physiological cognitive profiles, and can concurrently ameliorate negative neuropsychological status in healthy older adults.
Clare et al. [20] RCT (UK)	To evaluate a goal-setting intervention aimed at promoting increased cognitive and physical activity and improving mental and physical fitness, diet and health.	Three groups: control (IC)—an interview in which information about activities and health was discussed; goal-setting (GS)—an interview in which they set behaviour change goals relating to physical, cognitive and social activity, health and nutrition; and goal-setting with mentoring (GM)—the goal-setting interview followed by bi-monthly telephone mentoring. The one-to-one interviews lasted for 90 min.	12 months.	75 healthy elderly (IC—27 subjects; GS—24 subjects; GM—24 subjects); age: 50+ years.	The Lifetime of Experiences Questionnaire (LEQ), Physical Activities Scale for the Elderly (PASE), a battery of cognitive tests, audio-recordings of the interviews, statistical analysis.	The results show that at 12-month follow-up, the two goal-setting groups increased their level of physical (effect size 0.37) and cognitive (effect size 0.15) activity relative to controls.
Danthiir et al. [26] RCT (Australia)	To test whether docosahexaenoic acid (DHA)-rich fish oil slows 18-month cognitive decline in cognitively healthy individuals.	1720 mg DHA and 600 mg eicosapentaenoic acid or low-polyphenolic olive oil daily, as capsules in the intervention group and placebo in the control group.	18 months.	194 subjects in the intervention group and 196 in the control group; age: 65–90 years.	A battery of cognitive tests, statistical analysis.	The results show that supplementing older adults with fish oil does not prevent cognitive decline.
Kean et al. [12] RCT (UK)	To examine whether eight weeks of daily flavanone-rich orange juice consumption was beneficial for cognitive performance in healthy older people.	Daily 350 mg consumption of flavanone-rich 100% orange juice and equicaloric low-flavone (37 mg) orange flavoured cordial (500 mL).	Eight weeks	37 healthy subjects, mean age: 67 years.	A battery of cognitive, executive function and episodic memory tests, statistical analysis.	The results indicate that after the 8-week consumption of flavanone rich 100% orange juice, the global cognitive performance was significantly improved (*p* < 0.05).
Kulzow et al. [13] RCT (Germany)	To investigate the impact of omega-3 fatty acid supplementation on memory functions in healthy older adults.	Daily intake of 2200 mg long-chain polyunsaturated omega-3 fatty acids (LC-*n*3-FA) in the intervention group and placebo in the control group.	26 weeks.	22 healthy older subjects in the intervention group and 22 in the control group, age: 50–75 years.	Visuospatial object-location-memory task (LOCATO), standard neuropsychological tests, statistical analysis.	The findings reveal that the daily intake of LC-*n*3-FA has a positive impact on memory functions in healthy older people (*p* = 0.049).
Lehtisalo et al. [21] RCT (Finland)	To discuss the success of dietary counselling intervention among healthy older individuals.	Dietary intervention counselling: 3 individual and 8 group sessions.	Two years.	631 healthy older subjects in the intervention group and 629 in the control group, age: 60–77 years.	Food records, statistical analysis.	The findings show that the intake of several vitamins and minerals remained unchanged or increased in the intervention group and that the dietary counselling may have a positive impact on age-related diet quality and cognitive performance.
Mastroiacovo et al. [22] RCT (Italy)	To evaluate the effect of flavanol consumption on cognitive performance in cognitively intact elderly people.	A drink containing 993 mg high flavanol (HF), 520 mg intermediate flavanol (IF), or 48 mg low flavanol (LF) cocoa flavanols (CFs).	Eight weeks.	90 cognitively intact elderly subjects divided into three groups (HF, IF, LF); age: 61–85 years.	A battery of neuropsychological tests, blood pressure measures, statistical analysis.	The results reveal that regular CF consumption can reduce some measures of age-related cognitive dysfunction, possibly through an improvement in insulin sensitivity.
Nilsson et al. [23] RCT (Spain)	To evaluate effects on cognitive functions and cardiometabolic risk markers with a mixture of berries intervention in healthy older individuals.	Daily intake of 795 mg berry beverage with polyphenols or dietary fibre or 11 mg berry control beverage with no poly phenols or dietary fibre.	Five weeks.	20 healthy subjects in the intervention group and 20 healthy subjects in the control group, mean age: 50–70 years.	A battery of cognitive tests, cardiometabolic tests and statistical analysis.	The results indicate that the subjects performed better in the working memory test after the berry beverage compared to after the control beverage (*p* < 0.05).
Scott et al. [11] RCT (USA)	To explore the effect of the daily consumption of one avocado on cognition.	1 avocado daily in the intervention group and 1 potato or 1 cup of chickpeas in the control group.	Six months.	20 healthy subjects in the intervention group and 20 healthy subjects in the control group, mean age: 63 years.	A battery of cognitive tests, statistical analysis.	The results show that including the daily intake of one avocado may have a positive impact on cognitive performance in healthy older individuals, specifically on their working memory (*p* = 0.036) or sustained attention (*p* = 0.033).
Sindi et al. [24] RCT (Finland)	To assess whether baseline leukocyte telomere length (LTL) modified the cognitive benefits of a 2-year multidomain lifestyle intervention.	Participants were randomly assigned to the lifestyle intervention (diet, exercise, cognitive training, and vascular risk management) and control (general health advice) groups.	Two years.	775 healthy subjects (392 control, 383 intervention), at the age of 30–77 years.	A battery of neuropsychological tests, blood samples, statistical analysis.	The findings of the intervention reveal that cognitive benefits were more pronounced with shorter baseline LTL, particularly for executive functioning, indicating that the multi-domain lifestyle intervention was especially beneficial among higher-risk individuals.
Valls-Pedret et al. [10] RCT (Spain)	To investigate whether a Mediterranean diet supplemented with antioxidant-rich foods influences cognitive function compared with a control diet.	Participants were randomly assigned to a Mediterranean diet supplemented with extra virgin olive oil (1 L/week), a Mediterranean diet supplemented with mixed nuts (30 g/day), or a control diet (advice to reduce dietary fat).	Five years.	447 cognitively healthy volunteers (233 women (52.1%); mean age, 66.9 years); three groups: two intervention groups and one control group.	A neuropsychological test battery, statistical analysis.	In an older population, a Mediterranean diet supplemented with olive oil or nuts is associated with improved cognitive function.

RCT: randomized controlled trial.
